# Retinoid X Receptor: Cellular and Biochemical Roles of Nuclear Receptor with a Focus on Neuropathological Involvement

**DOI:** 10.1007/s12035-021-02709-y

**Published:** 2022-01-11

**Authors:** Samridhi Sharma, Ting Shen, Nitin Chitranshi, Veer Gupta, Devaraj Basavarajappa, Soumalya Sarkar, Mehdi Mirzaei, Yuyi You, Wojciech Krezel, Stuart L. Graham, Vivek Gupta

**Affiliations:** 1 Macquarie Medical School, Faculty of Medicine, Health and Human Sciences, Macquarie University, Sydney, NSW Australia; 2 School of Medicine, Deakin University, Melbourne, VIC Australia; 3 Save Sight Institute, University of Sydney, Sydney, NSW Australia; 4 Institut de Génétique Et de Biologie Moléculaire Et Cellulaire, INSERM U1258, CNRS UMR 7104, Unistra, 67404 Illkirch-Graffenstaden, France

**Keywords:** Nuclear receptors, Retinoid X receptor (RXR), Lipid X receptor (LXR), Peroxisome proliferator-activated receptor (PPAR), Nuclear receptor-related 1 (Nurr1), Heterodimerisation, Neuronal stress, Neuroinflammation, Neuroprotection, Glucose metabolism, Lipid metabolism ligand, Endogenous ligands, Exogenous ligands, Bexarotene, Glaucoma, Alzheimer disease, Parkinson disease, Multiple sclerosis, Stroke

## Abstract

Retinoid X receptors (RXRs) present a subgroup of the nuclear receptor superfamily with particularly high evolutionary conservation of ligand binding domain. The receptor exists in α, β, and γ isotypes that form homo-/heterodimeric complexes with other permissive and non-permissive receptors. While research has identified the biochemical roles of several nuclear receptor family members, the roles of RXRs in various neurological disorders remain relatively under-investigated. RXR acts as ligand-regulated transcription factor, modulating the expression of genes that plays a critical role in mediating several developmental, metabolic, and biochemical processes. Cumulative evidence indicates that abnormal RXR signalling affects neuronal stress and neuroinflammatory networks in several neuropathological conditions. Protective effects of targeting RXRs through pharmacological ligands have been established in various cell and animal models of neuronal injury including Alzheimer disease, Parkinson disease, glaucoma, multiple sclerosis, and stroke. This review summarises the existing knowledge about the roles of RXR, its interacting partners, and ligands in CNS disorders. Future research will determine the importance of structural and functional heterogeneity amongst various RXR isotypes as well as elucidate functional links between RXR homo- or heterodimers and specific physiological conditions to increase drug targeting efficiency in pathological conditions.

## Introduction

Most cells express receptor proteins that recognise and respond to diverse internal and external stimuli. By acting as transcription factors, the nuclear receptors play a central role in regulating many biological processes, ranging from gene expression, maintenance of intracellular metabolic and physiological balance to cellular differentiation and development [1]. The nuclear receptor superfamily is comprised of 48 members of receptor/proteins, and amongst these, retinoid X receptor (RXR) is one of the most intriguing member that plays a role in regulating diverse physiological and disease processes [2]. RXRs are involved in transcriptional regulation by forming obligate heterodimers with multiple nuclear receptors, although activity of homodimer and homotetramers has also been suggested [3, 4]. Due to this inherent capability to engage with different nuclear receptors, RXR and its partners orchestrate pleiotropic physiological and molecular roles in cell and tissue-specific manner [2, 3]. Studies attempting to unravel complexity of the RXR-dependent transcriptional regulation have demonstrated RXR’s ability to regulate transcription in a ligand-dependent or ligand-independent manner [5, 6]. This dual regulatory feature effectively means that RXR partners can be divided into permissive and non-permissive heterodimers [6]. The heterodimer activation induced by RXR agonist is classified under the permissive category and a non-permissive category corresponding to those heterodimers which are activated by ligands specific to the other nuclear receptors that partner with RXR, participating in a silent or supportive manner [6]. Permissive partner heterodimerisation in which both RXR and its partner ligand are simultaneously activated confers a synergistic biological response, and such coupling to achieve a biochemical function is necessary in a variety of physiological and pharmacological milieus [5]. RXRs as paralogues are ubiquitously expressed including in the central nervous system (CNS) and peripheral nervous system (PNS) although specific isotypes display more restricted expression patterns [3, 7].

Through their ligand-activated heterodimeric complexes, RXRs regulate transcription of genes that play critical role in cellular differentiation and metabolic processes including lipid and glucose metabolism in neurons [2, 3, 8]. RXR ligands such as bexarotene or honokiol have been extensively studied and demonstrated to exhibit neuroprotective effects in animal models of glaucoma, Alzheimer disease (AD), and Parkinson disease (PD) [9–12]. The receptors are also implicated in microglial activation to protect tissues against neuroinflammatory response and mediate clearance of debris in multiple sclerosis (MS) and brain stroke pathology although involvement of specific homo- or heterodimers with other partners need to be further investigated [13, 14]. This diverse RXR functional spectrum is attributed to the fact that RXRs are positioned centrally in the nuclear receptor signalling network interacting directly and indirectly with transcriptional machinery frequently in a ligand-dependent manner [3]. As our understanding of RXRs and their heterodimers in regulating transcription, homeostasis, neuronal differentiation, neuroinflammation, and neuroprotective effects has expanded in recent years, RXRs have emerged as a promising therapeutic target in neurological disorders. This review discusses our current understanding of the structure, ligands, interactions, and functions of RXR. We also discuss the involvement of RXRs in various neuronal disorders and the potential therapeutic prospects of modulating the receptor function. The review closes with a brief discussion of on-going clinical trials and provides future perspectives of RXR and its partners in neurological research.

## Historical Importance and Positioning of RXR in Nuclear Receptor Superfamily

In 1990, the molecular cloning and transcriptional activation studies performed by Mangelsdorf and co-workers identified RXR that responded specifically to the vitamin A metabolites [15]. RXR differed in structure from the previously identified transcriptional regulator retinoic acid receptor (RAR) [15] but had an analogous mechanism of transcriptional regulation with that of steroid and thyroid hormone receptors (THR) [15]. Further characterisation of RXR and its activation by 9-cis retinoic acid (9-cis-RA) but not all-trans RA, the latter being an endogenous ligand of RARs, resulted in recognition of a new retinoid response pathway that not only differed from RAR in response to retinoids but was also evolutionarily conserved [15] and demonstrated ability to heterodimerise with multiple receptors [5, 6]. Due to this reason, RXR was established as one of the founding members of the “adopted” orphan class of nuclear receptors [5]. Mangelsdorf and co-workers (2014) called the discovery of the ligand activation mechanism of the RXR and its ability to heterodimerise with other nuclear receptors as a “big bang” discovery [5]. Although 9-cis-RA was referenced for a long time as potential physiological, endogenous ligand of RXRs, its detection in physiological conditions was questionable leading in 2015 to the identification of 9-cis-13,14-dihydroretinoic acid (9-cis-DHRA) as the first endogenous retinoid acting as RXR agonist [16]. Various dietary lipid metabolites such as fatty acids, bile acids, and xenobiotic lipids have also been characterised as ligands for RXR-partnered orphan receptors [17, 18].

Currently, the nuclear receptor superfamily is comprised of 48 members in humans although this number may vary across different species, for example, teleost zebrafish has been reported to have 73 nuclear receptor members [2, 19, 20]. In metazoan physiology, nuclear receptors modulate gene expression in response to small lipid-based molecules such as natural hormones, lipids, bile acids, or synthetic ligands [21]. Ligand binding induces conformational changes in the receptor and stimulates a cascade of downstream events that direct the nuclear receptor to the DNA transcriptional regulation sites [22]. The nuclear receptors are divided into four types based on the mechanisms in which nuclear receptors bind to their respective ligands, attributing RXR the highest promiscuity amongst nuclear receptor superfamily. A detailed review by Evans and co-workers (2014) described binding ability of RXR’s permissive and non-permissive partners and their corresponding pleiotropic functional roles [5]. RXR forms heterodimers or tetramers with the ligand-activated partners and plays crucial roles in regulating cellular pathophysiology [5].

## Molecular Structure of the RXR

**Fig. 1:**
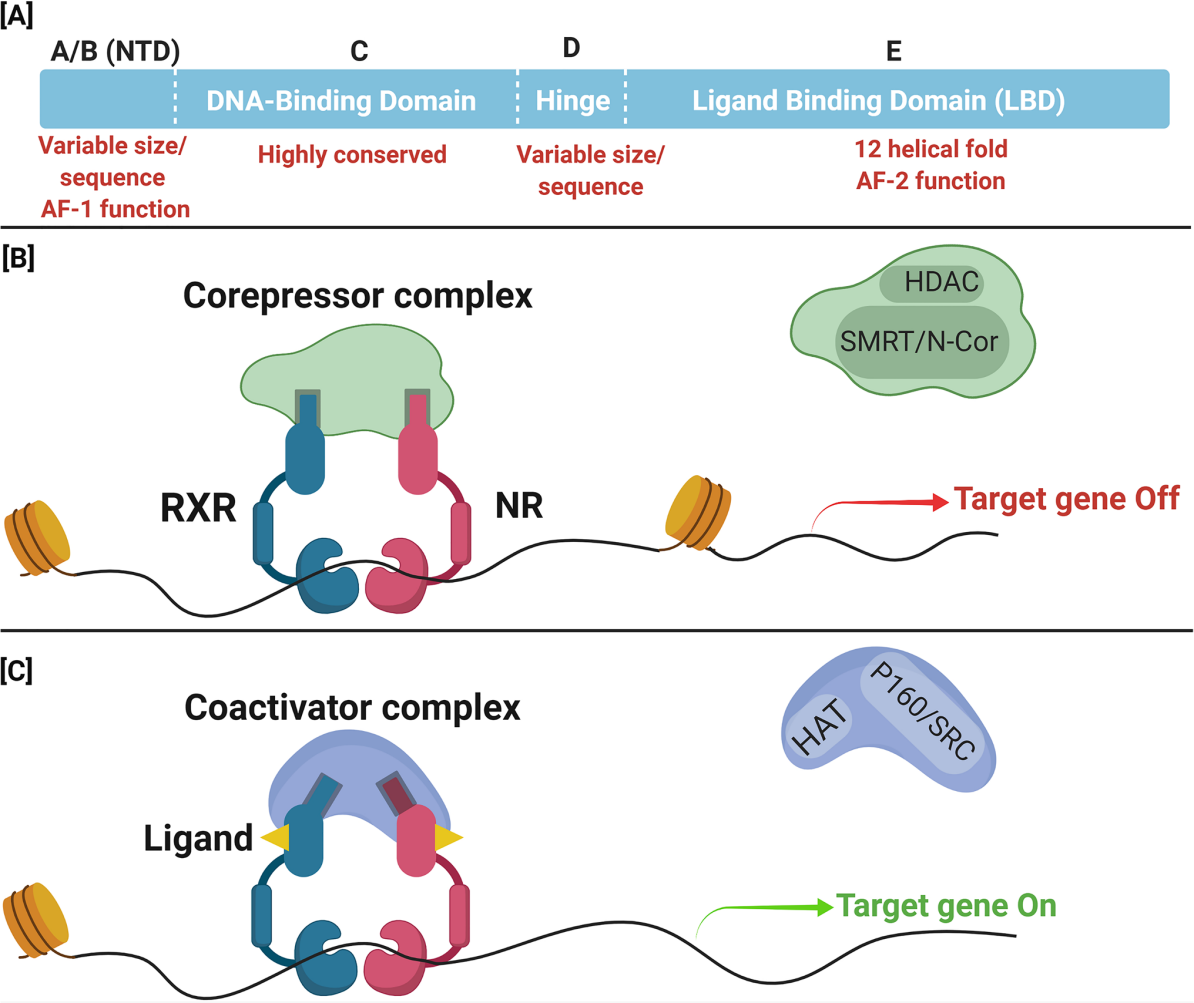
Structure of RXR and its transactivation, **A** Schematic representation showing six functional domains of RXR, which include N-terminal activation function 1 (AF1), DNA-binding, ligand-binding, and C-terminal AF2 domains. RXR heterodimers binds with the response element of the promoter region of the target gene. **B** In the absence of agonist ligand, AF-2 domain promotes interaction with corepressor complex [histone deacetylase (HDAC) and silencing mediator for retinoid and thyroid receptors/nuclear receptor corepressor (SMRT/N-Cor)] which blocks the transcription process. **C** Ligand interaction may cause conformational changes in the AF2 domain, which destabilises corepressor interaction and promotes coactivator [histone acetyltransferases (HAT) and p160/steroid receptor coactivator (P160/SRC)] binding leading to activation of target gene transcription process

Similar to the other nuclear receptors, human RXR (Uniprot no. P19793) protein structure consists of multiple functional domains [17]. These domains include a poorly conserved and variable length N-terminal domain (NTD) (domain A/B) [3, 5] (Fig. 1A) which includes a ligand-independent activation coactivator binding region AF1 [3, 17]. There is also a highly conserved DNA-binding domain (DBD) that contains two zinc-finger motifs (domain C), which mediate RXR binding to specific DNA sequences [3, 17]. A flexible hinge (domain D) connects the DNA and ligand binding domain (LBD) (domain E) [3, 17] (Fig. 1A). LBD is highly conserved and contains activation function (AF-2) sequence to which the coactivators (CoAs) or corepressors (CoRs) bind to regulate the transcription activation [3, 17] (Fig. 1B, C). The DBD comprises of two zinc-finger domains that bind and interact with the phosphate backbone and nucleotides of the DNA major groove facilitating DNA dimerisation [23]. The hinge area next to the DBD constitutes of approximately 201–229 residues in human RXRs. This area provides flexibility to the RXR-LBD region so that it accommodates LBD motif from other nuclear receptors such as from PPAR. RXR crystallographic studies have further elucidated the molecular basis of heterodimeric interactions of RXR [22, 24–26]. The biophysical nature of RXR and its heterodimers was also discussed by Nagy and co-workers (2004) [27]. Essential for such interactions is the ligand binding domain which activates the bound receptors into potent activators of transcription [3, 28].

To understand the heterodimerisation ability of RXR and its transactivation of target genes, we need to understand the DNA sequence-specificity for RXR binding [29]. Classical regulatory elements recognised by RXR heterodimers consist of two direct repeat (DR) half sites separated by 2, 3, 4, or more nucleotides (e.g. 5′-AGGTCA-N_x_-AGGTCA-3′) to form DR2, DR3, or DR4 binding sites, whereas in homodimerisation process, RXR binds to DRs separated by one nucleotide called DR1 [23, 30]. DR1 requires the sequential introduction of single nucleotides within the spacer of two DR1 half sites, in turn, generating novel binding motifs such as DR2, DR3, DR4, and so on. [30]. This flexibility due to the innate structure of the RXR-LBD allows it to adopt various conformations that lead to its heterodimerisation ability with PPAR, LXR, FXR, PXR, and CAR receptors [30]. In the case of the RXR/PPAR, the heterodimer preferentially binds to PPAR response element, which consists of two half sites separated by single nucleotide [23]. Due to this molecular plasticity, each heterodimer can occupy one of the half sites, while RXR continues to bind to the remaining moiety [5]. This DR1 sequence was suggested as an important determinant that has likely played a role in molecular evolution of RXRs [5]. It grants RXR flexibility in its ligand binding ability with multiple partners as the interactions are not hardwired and are dependent on the DNA-binding sites that can undergo induced fit adjustment to allow the dimerisation process [5, 31].

## RXR Isotypes and Their Expression

In mammals, RXR exists in three isotypes, RXRα, β, and γ, also known as NR2B1, 2, and 3 encoded by distinct genes (9q34.2, 6p21.32, 1q23.3 in human) and on mouse chromosomes 2, 17, and 1, respectively [15, 32]. Human RXRα, ß, and γ share 97.4%, 94.75%, and 98.27% homology, respectively, with their mouse counterparts in their ligand binding domain sequence (225–462) [32].

### RXRα

This protein belongs to nuclear receptor subfamily 2 group B member 1 and human RXRα comprises of an open reading frame of 462 amino acids with molecular mass 50,811 Da [15]. It has been identified to be localised in the nucleus, cytoplasm, and mitochondria [32]. RXRα is a predominant isotype expressed in liver, kidney, and brain tissues. Alternate splicing may lead to further heterogeneity by generating different isotypes: RXRα1, α2, α3, and α4 [33]. RXRα1 is the major isotype expressed in most tissues, while RXRα2 and RXRα3 have been identified in the testis and RXRα4 in not yet characterised functionally [33].

### RXRβ

Human RXRβ is localised on chromosome 6p21.32 proximal to HLA class II gene [34]. It is specifically expressed in the endothelial cells and monocytes and has been detected in multiple cancer cell lines [32]. The human homologue of mouse β1 isoform, RXRβ2, is largely conserved across species with two ATG initiator codons that could produce N-terminal variants [35]. The third isoform mouse RXRβ2E contains an SLSR (a four amino acid sequence) in the ligand binding domain that was shown to negatively impact transactivation process [36]. In the mouse CNS, RXRβ is semi-ubiquitously expressed with different contribution of different isoforms depending on brain region [7, 37].

### RXRγ

This gene is 14 kb long comprising nine introns with size ranging from 107 base pairs to more than 30 kb. mRXRγ1 gene product is encoded by 10 exons, while mRXRγ2 is transcribed from and alternative promoted on the 3rd exon [32, 38]. mRXRγ1 is highly expressed in muscle and brain tissues, while mRXRγ2 is expressed predominantly in the skeletal and cardiac muscle [38]. Expression of RXRγ in mouse brain is very high but restricted only to few brain regions including the striatum, dispersed cells within frontal and entorhinal cortex, hippocampus, and amygdala [7, 39]. Further, RXRγ^−/−^ knockout BALBcByJ mice display memory deficits and depression-like behaviour [40].

The fine-tuning of RXR transcriptional activity is also affected by post-translational modifications (PTMs) [41]. The phosphorylation of human RXRα at Ser 260 via activated Ras-Raf-MAPK cascade modulates CoA recruitment to the RXR/VDR heterodimers [41]. RXR phosphorylation on distinct Ser residues regulates its cooperation with RAR, and this PTM has been demonstrated to be relevant for both the transcription of the retinoic acid target genes and degradation of the receptors [42]. These specific effects of particular PTMs on RXR activity underscore the flexibility of receptor structure–function relationship and emphasises PTM roles in regulating RXR signalling [41]. However, the epigenetic landscape of RXR and its interactions continues to be understudied.

## Understanding RXR Evolutionary Conservation in Context of Ligand Binding Function

RXR have been recognised across most metazoan species, with the earliest RXR orthologue documented in *Trichoplax adhaerens* (Ta) [43, 44]. Similar to the case with nuclear receptors in general, RXRs have not been identified in fungi, plants, or unicellular eukaryotes [45]. Ta RXR shares 81% of homology with DBD and 70% with LBD of the human RXR. The DNA-binding motif specificity is conserved up to 85% with that of human RXRα [46].

More recently, 9-cis-DHRA, an endogenous physiological RXR ligand, identified in 2015, has been suggested to be a prototype ancestral retinoid that evolved to be a RXR ligand in vertebrates [16, 47, 48]. Importantly, this ligand is an active form of vitamin A5/X, a new class of vitamin A comprising 9-cis-13,14-dihydroretinol and 9-cis-13,14-dihydro-β, β-carotene as nutritional precursors which are distinct from those known for classical vitamin A1 pathway defined by all-trans β, β-carotene, and all-trans retinoids [39]. Understanding the metabolism of 9-cis-DHRA and its precursors in different species together with their nutritional availability in species-respective environments will help trace the relevance of this intriguing ligand and help understand the genetic and environmental interplay in shaping the ligand–receptor interactions of RXR [49].

## RXR Interacting Partners and Ligands

A very high evolutionary conservation of RXR-LBD has resulted in identical ligand binding pocket structure amongst all three RXR paralogs in human and mouse. This suggests similar ligand specificity and possibly overlapping protein interaction partners amongst different isotypes [3]. The involvement of LBD, both in ligand binding and interactions with other receptors and coregulators, not only allows multiple partners for RXR but also serves as a perfect model to understand the receptor structure–activity relationship [48]. Deciphering the dynamics of RXR with its interacting partners will facilitate understanding of their biochemical and signalling roles. The list of RXR protein partners includes (i) transcription factors, (ii) transcriptional cofactors including CoAs and CoRs, (iii) DNA-modifying agents and, and (iv) other proteins in its subcellular vicinity [3]. RXR interactions with multiple protein partners underlie pleiotropic effects in multiple biological processes, but the true extent and significance of these interactions in various physiological and disease conditions remain to be elucidated [3]. Importantly, RXR ligands may differentially affect activities of different RXR heterodimers [45]. This section will discuss both endogenous ligands of RXR and exogenous ligands reported from natural sources or prepared synthetically.

### Endogenous Ligands

Naturally occurring ligands, such as endogenous or nutritional, bind to RXRs and may serve as molecular regulator of various physiological functions [50]. The search for an endogenous ligand for RXR has been technically challenging. For a long time, 9-cis-RA was suggested as an endogenously occurring functionally relevant RXR ligand; however, a number of studies reported that in physiological conditions, 9-cis-RA was either not detected or was present at concentrations insufficient to bind and activate RXR-mediated signalling [16]. Accordingly, 9-cis-RA has been detected in the range of 0.03 to 0.003 nM in various tissues [47, 51]; however the minimal concentration required for transactivation of RXR is 10–100 nM [16, 52]. Therefore, such low levels may not be sufficient to induce RXR transactivation in vivo, minimising the likelihood of 9-cis-RA acting as endogenous physiological ligand for RXR [47, 53, 54].

Using LC–MS/MS and dedicated standards, Rühl and co-workers (2015) identified the presence of 9-cis-DHRA in mouse liver (∼450 nM), serum (∼400 nM), and brain (∼130 nM) tissues with concentrations sufficient to induce and maintain RXR-dependent activities [16, 48]. Crystallographic studies established that 9-cis-DHRA binds to the RXRα-LBD in a mechanism similar to that reported for 9-cis-RA and triggers RXR transactivation at about 100 nM levels [16]. In monocyte-derived human dendritic cells, 9-cis-DHRA also transactivated target genes in the heterodimer complexes LXR/RXR, PPAR/RXR, and RAR/RXR [16]. Reduced levels of 9-cis-DHRA were observed in Rbp1^−/−^ (cellular retinol binding protein) mice brains, liver, and plasma reflecting on the compromised RXR-mediated signalling in the absence of this protein, leading to memory deficits [16]. Further studies may unravel the biochemical roles and pathophysiological relevance of the 9-cis-DHRA in the CNS. Thus, 9-cis-RA and endogenous 9-cis-DHRA may be relevant for the activation of permissive RXR heterodimers including also RAR/RXR heterodimers as both retinoids can bind and transactivate RARs in nanomolar range [55].

Fatty acids constitute an important category of RXR ligands. A series of the free fatty acids (FFAs) including in particular docosahexaenoic acid (DHA) or eicosapentaenoic acid (EPA) can bind RXRs with varying affinities and induce their transcriptional activation at different concentrations [56]. The efficiency of DHA in transactivation of RXR influencing neuronal function was best shown at 10 µM concentration compared to other FFAs like docosatetraenoic, oleic, and arachidonic acids that may activate RXR at 50–100 µM concentrations [56]. DHA can activate RXRα and induce neurite outgrowth in neuronal cells at low concentration [57]. Arachidonic acid is also shown to activate the RXRα isoform but with lower efficacy [57]. Accordingly, DHA in retina was shown to promote survival of rat photoreceptors upon H_2_O_2_ or paraquat-induced oxidative stress through activation of RXRs and the ERK/MAPK signalling pathway [58]. Another branched chain FFA, phytanic acid, demonstrated binding and activation of RXRα similarly to 9-cis-RA in rodents [59] but failed to induce RXR activation in human cell lines [60]. Phytanic acid concentration greater by three order of magnitude compared to that of 9-cis-RA was required to elicit similar effects [61]. Requirement of high concentrations of these diverse fatty acids or their metabolites for activation of RXR-mediated signalling makes them good candidates for direct or indirect nutritional ligands of RXRs, relevant for modulating RXR activities after nutritionally induced rise in their concentration but might be less relevant for physiological due to insufficient concentrations [48].

### Exogenous Ligands

#### ***Naturally Occurring Ligands***

Several bioactive compounds isolated from traditional medicinal plant sources have been identified as RXR ligands. The first of such natural compound identified was honokiol, which was isolated from genus *Magnolia* and has been extensively studied in its relevance to the neurological disorders [62]. Honokiol activates the apolipoprotein E (ApoE)/ATP binding cassette transporter1 (ABCA1) expression through RXR-dependent mechanism [62]. The compound was shown to regulate cholesterol metabolism and homoeostasis by direct activation of the LXR/RXRβ in murine astrocytes and in neuronal culture at EC_50_ ∼10 μM [62]. In addition to its roles in lipid metabolism, honokiol also regulates glucose uptake by cells and activates the PPARγ/RXR signalling by directly binding to the PPARγ [63]. Honokiol has also shown neuroprotective and anti-neuroinflammatory effects in AD and PD, but whether this activity is mediated via RXR activation remains to be established [64, 65]. Another ligand, magnolol, also isolated from *Magnolia officinalis*, has been shown to interact with RXRα and PPARγ with EC_50_∼40 μM and ∼2 μM, respectively, showing its preference for activation of PPARγ/RXR heterodimers at EC_50_∼10 μM [66].

There are a few more naturally identified ligands of RXR such as drupanin, isolated from Brazilian green propolis with selective affinity towards PPARγ/RXR heterodimer and capable of performing transactivation at EC_50_ of 2–7 μM [67]. Flavanones isolated from *Sophora tonkinensis* also showed specific agnostic activity for RXR and activated direct target genes of RXR/LXR, RXR/PPARβ/δ, and PPARγ complexes [68]. Bigelovin, a sesquiterpene lactone isolated *Inula hupehensis*, binds specifically to RXRα-LBD with demonstrated anti-carcinogenic properties as observed in colon cancer cells [69]. Crystallographic evidence has shown that RXR-LBD binding mechanism with bigelovin is remarkably distinct from that observed with other RXR ligands [69]. This might explain limited capability of the ligand to activate FXR/RXRα heterodimer complex while inducing transactivation of other heterodimeric complexes [66]. More recently, valerenic acid, a sesquiterpenoid, isolated from *Valeriana officinalis* has been shown to bind and activate RXR [70]. Valerenic acid is a highly specific ligand of RXR with no affinity towards PPAR, LXR, RAR, FXR, VDR, PXR, and CAR receptors [70]. Valerenic acid binding to RXR was shown to efficiently transactivate RXR target genes ABCA1 and ApoE at EC_50_ = 5 μM for RXRß, EC_50_ = 27 μM for RXRα, and EC_50_ = 43 μM for RXRγ isoforms [70]. Further pharmacokinetic and pharmacodynamic studies will establish the therapeutic potential of these naturally occurring RXR ligands in various health and disease processes.

#### ***Synthetic Ligands***

Several structural mimetics of the naturally occurring RXR ligands have been identified that interact with RXR-LBD domain and regulate its activity. Here, we discuss some of the potent synthetic ligands that have been shown to interact with RXR and exert neurological effects. Bexarotene (LGD1069) is a pan-RXR agonist and an approved US-FDA anticancer treatment for cutaneous T cell lymphoma [71]. The drug selectively binds to RXR α, β, and γ isotypes with high affinity as demonstrated by *K*_d_ values of 14 ± 3 nM; 21 ± 4 nM; and 29 ± 7 nM, respectively, in comparison to RAR (*K*_d_ > 1000 nM) [72]. Chitranshi and co-workers (2019) reported on binding affinity of RXR agonists to RXR using molecular docking and computational simulation techniques [73]. Dynamic simulation studies confirmed that the hydrogen bonding and hydrophobic interactions were highly stable in all the three isoforms of the RXR–bexarotene complex showing Ala and Arg (Ala327 and Arg316 in RXRα; Arg387 and Ala398 in RXRβ; and Arg95 and Ala106 in RXRγ) as key interacting residues when compared to other RXR agonists such as RA, calcitriol, tamibarotene, and acitretin [73]. Bexarotene showed high binding with the RXRα and β compared to the RA, while RXRγ-LBD displayed weaker affinity [73]. Biological assays demonstrated that RXR–bexarotene interactions promote neurite growth in SH-SY5Y neuronal cells [73]. Importantly, this study suggested that RXRγ–bexarotene complex is rather unstable in comparison to the RXRα/ß complex formation, supporting that further pharmacodynamic investigations are required to understand the differential effects of bexarotene on RXR isotypes in neurodegenerative disorders [73]. In the PD murine model, bexarotene mediated activation of Nurr1/RXR heterodimer complex imparting neuroprotective effects and restored behavioural functions [8]. Recently, our group demonstrated the ability of bexarotene to induce RXR activation in the neuronal cells and retinal tissues [9, 10]. Bexarotene treatment (0.1 µM) protected the neuronal cells against Aβ-induced endoplasmic reticulum (ER) stress and suppressed pro-apoptotic BAD protein activation while promoting neurite outgrowth [10]. At higher concentrations, bexarotene (10 µM) showed an upregulation of neurotoxic effects which was prevented by inducing pharmacological activation of the tropomyosin receptor kinase B (TrkB) receptor signalling [9, 10]. In another study, bexarotene reduced the levels of soluble Aβ in the brain interstitial fluid by 25% in 2-month-old APP/PS1 mice via RXR activation [74]. The administration of bexarotene has been shown to enhance clearance of soluble Aβ from the brain within hours in an ApoE-dependent manner in a mouse model of AD, providing a rationale to develop this anticancer drug as a potential AD therapeutic agent [75]. Bexarotene-activated RXR also induces expression of oestrogen-synthesising enzymes in hippocampal slice cultures [76]. The structural analogue of bexarotene, LG100268, was found to exhibit much higher affinity for RXR with Kd of 3 nM. LG100268 caused transactivation of RXR at concentration that is at least tenfold lower than that of 9-cis-RA [72]. Treatment with this drug was also observed to induce several target genes during monocyte differentiation into dendritic cells or during osteoclast differentiation by activation of RXR homodimers as indicated by transcriptome profiling [77]. The compound was also demonstrated to activate LXR/RXR, and the PPARγ/RXR heterodimers and downstream consequences of their actions need to be studied for better understanding of its mode-of-mechanism [78].

Other ligands with varying levels of interaction affinity and impact on RXR conformation such as CBt-PMN have been reported [79]. This pharmacophore selectively binds and activates heterodimers of LXRα/RXRα and PPARγ/RXRα in vitro (EC_50_ = 143 nM) and in vivo (EC_50_ = 15 nM) [79]. RXR activation using CBt-PMN was implicated in providing beneficial effects in mediating glucose regulation in animal models of type 2 diabetes [79]. Further, chemical library screening experiments revealed that XCT0135908 was able to selectively activate the Nurr1/RXR heterodimers which could find applications in the preservation of nigrostriatal dopamine system in PD [80]. Similar effects have been reported for other selective activator of Nurr1/RXR, such as HX600 [81]. In addition, inhibitors of sterol biosynthesis, such as pitavastatin, fluvastatin, and their analogues, demonstrate varying levels of RXRα antagonism [82]. Rosiglitazone and domperidone were shown to exhibit RXR agonistic action in the presence of 9-cis-RA, wherein these compounds activated PPAR/RXR heterodimer complex and modulated glucose metabolism [82]. Furthermore, treatment with methoprene acid (MPA) which is a pesticide was shown to induce transcription at RXR-specific CRBPII promoter for all three RXR isotypes with EC_50_ of 2 μM in insect and 20 μM in mammalian cell lines, respectively [83]. Its parent molecule, methoprene, also induced upregulation of the target genes ABCA1 and ABCG1 implicated in regulating cholesterol efflux in rat astrocytes [83]. Altogether, these endogenous and exogenous ligands of RXR exhibit a range of biochemical effects and modulate various metabolic processes under both physiological and disease conditions.

## RXR Involvement in Various Biochemical Networks

RXRs are crucial regulators of macrophage lipid metabolism, monocyte migration, ApoE synthesis, and cholesterol homeostasis in astrocytes, regulating inflammatory responses of microglia and oligodendrocyte progenitor cell differentiation [4, 84–87]. This involvement of RXR in multiple biochemical networks is concomitant with its ubiquitous expression in tissues such as the liver, kidneys, small intestine, cardiac myocytes, monocytes, macrophages, Kupffer cells, adipose tissue, and colon mucosa [88–94]. This section will elucidate the roles of the RXR and its heterodimers in various biochemical and metabolic networks.

### Role of RXR and Its Partners in Reducing Cellular Stress and Neuroinflammation

**Fig. 2:**
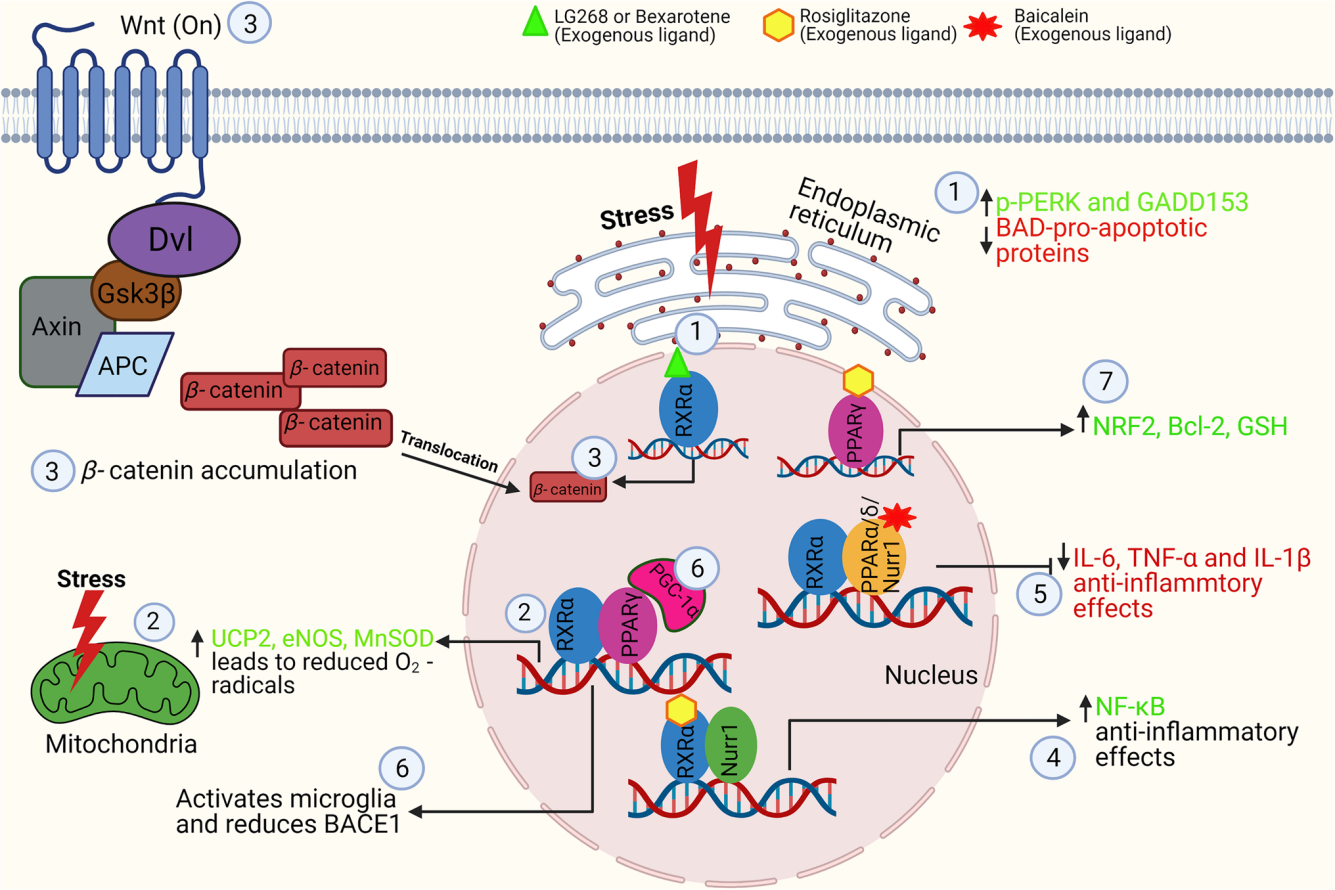
Schematic representation of RXR and its partners in reducing neuronal stress and neuroinflammatory effects: RXR stimulation by exogenous ligand bexarotene reduces the endoplasmic reticulum stress markers p-PERK and GADD153 in retinal ganglion cells and supresses BAD protein activation (**1**). Activation of the RXRα/PPARγ heterodimer with ligands activates proteins UCP2, eNOS, and MnSOD to reduce mitochondrial stress (**2**). The RXRα heterodimer regulates transcription of β-catenin (**3**). LG268 and bexarotene binds to RXRα/PPARα/β and Nurr1 forming a heterodimer complex that inhibits TNF-α, IL-1β, and IL6 and promotes NF-κB anti-inflammatory effects in microglia and macrophages (**4**, **5**). A PPARγ-coactivator-1α (PGC-1α) reduces expression of BACE-1, a β-amyloid (Aβ) precursor protein cleaving enzyme (**6**). PPARγ increases the expression antioxidants GSH, NRF2, and Bcl-2 to play a protective role against apoptosis (**7**)

RXR heterodimerises with its permissive partners modulate unfolded protein response (UPR) and other ER stress pathways [95]. Treatment with RXR agonist bexarotene was shown to reduce the expression of p-PERK and GADD153 ER markers in SH-SY5Y cells treated with neurotoxic *β*-amyloid (Aβ) in a dose-dependent manner [10]. Treatment with low concentrations of bexarotene also alleviated the expression of pro-apoptotic BAD proteins in AD mouse model [10] (Fig. 2 (1)). In another study conducted by our group, treatment with bexarotene protected retinal ganglion cells in glaucoma mouse models that were associated with reduction in expression of ER stress markers [9]. The ligand-activated PPARγ/RXR has previously been demonstrated to alleviate mitochondrial stress response accompanied by upregulation of UCP2, eNOS, and MnSOD scavenger proteins and reduced O_2_^−^ radical accumulation in the mitochondria [96, 97] (Fig. 2 (2)). Nurr1 is highly expressed in the CNS forming heterodimers with RXRs, and its activation is associated with RXR ligand LG268-induced neuroprotection in ventral midbrain cultures [80]. Nurr1 transcription complex activation is involved in midbrain dopaminergic (DA) and serotonergic neurogenesis along with activation of PITX3 and Wnt/*β*-catenin signalling [98, 99]. Separately, RXRα also inhibits the transcription of *ß*-catenin and directly regulates the Wnt/*β-*catenin cascade in colon cancer cells suggesting RXRα–*ß-*catenin axis as a potential therapeutic target [94] (Fig. 2 (3)). Dickey and co-workers (2017) demonstrated that bexarotene may reverse impaired oxidative metabolism and rescue altered mitochondrial morphology in neuronal cells derived from N171-82Q Huntington disease mouse model [100]. The agonist treatment resulted in improved proteostasis by inducing autophagy via its effects on PPARδ activation and controlling overall cellular quality control to achieve neuroprotection [100].

RXR heterodimerisation with PPAR, LXR, and Nurr1 also plays a role in regulating adaptive immune response and macrophage, monocyte, dendritic cell, and T cell functions through clearance of dead cells, T cell differentiation, and regulating gene repression of inflammatory mediators [101, 102]. Nurr1 was demonstrated to inhibit pro-inflammatory genes in LPS treated astrocytic and microglial cultures through NF-κB modulation [103] (Fig. 2 (4)). RXRα independently and in RXR/PPARδ complex influences innate inflammatory response by modulatory effects on cytokine and chemokine expression in myeloid cells and monocytes/macrophages [104, 105] (Fig. 2 (5)). Similar inhibition of the pro-inflammatory cytokines achieved through activation of LXR/RXR, PPARα/RXRα, and STAT3 pathways in inflammation-induced corneal angiogenesis has been reported to impart protection against inflammatory changes in rat cornea [106]. Baicalein (5,6,7-trihydroxyflavone), a flavonoid, was shown to enhance the PPARδ expression in brain microglia and suppress MAPK and NF-κB signalling resulting in augmentation of anti-inflammatory effects [107]. Similar attenuation of the neuroinflammation mediated by bexarotene has been implicated in rat model of subarachnoid haemorrhage [108]. These anti-inflammatory actions were shown to be mediated through its regulatory effects on PPARγ, SIRT6, and FoxO3A [108]. In addition, PPARγ-coactivator-1α (PGC-1α) which is a co-factor for transcription was implicated in the amelioration of microglial activation and reduced expression of amyloid precursor protein cleaving enzyme (BACE1) in APP23 AD mouse model [109, 110] (Fig. 2 (6)). RXR-activated PPARγ regulates oxidative stress response and prevents autophagy in association with increased expression of NRF2 in vivo in status epilepticus (SE) [111]. It was shown that PPARγ/RXR complex upon binding to its agonist rosiglitazone provided significant protection against hippocampal neuronal loss in epileptic rat model, attributed to enhanced expression of antioxidant GSH [111, 112] (Fig. 2 (7)). The overexpression of the PPARγ also led to increased expression of anti-apoptotic protein Bcl-2, leading to mitochondrial stabilisation in hippocampal neurons in vivo [113, 114]. Collectively, there is ample evidence to support the involvement of PPARγ in neuroprotection upon heterodimer formation with RXR [115]; however, direct RXR role in imparting neuroprotection in hippocampal and glial cells remains to be established.

### RXR and Its Partners in Regulating Lipid Metabolism

**Fig. 3:**
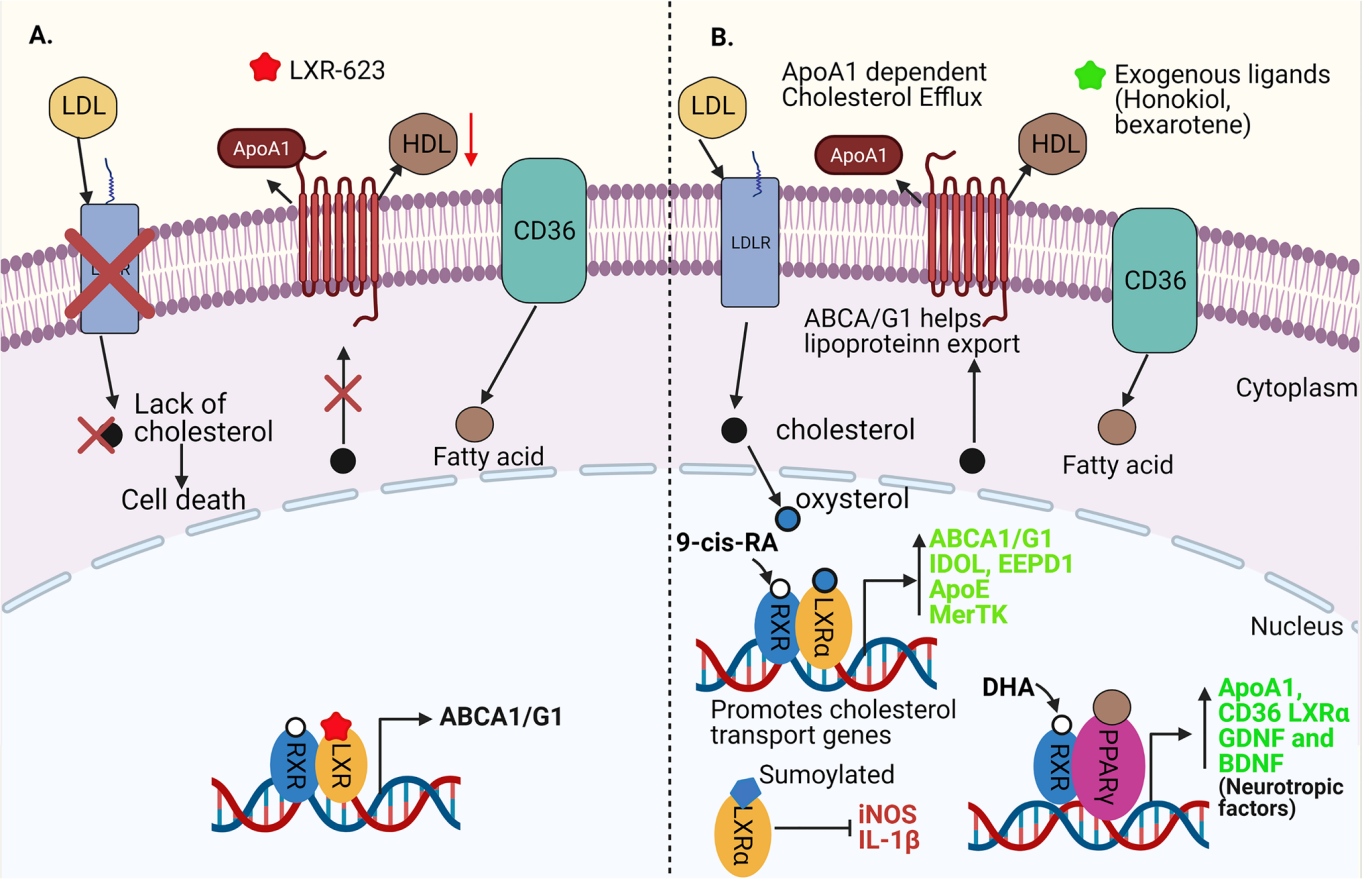
RXR and its heterodimers in transcriptional regulation of cholesterol homeostasis and lipid metabolism.** A** Representative RXR/LXRα activation in glioblastoma (GBM) cells **—** GBM cells upregulate LDLR to increase the cholesterol uptake necessary for their survival which in turn supresses the de novo synthesis of cholesterol. Activation of the RXR/LXRα heterodimer by an exogenous agonist LXR-623 increases transcription of ABCA1, a major transporter of cholesterol carrying lipoproteins between neurons and glial cells but incapable of de novo synthesis of the cholesterol; GBM cells upon treatment with LXR-623 undergo significant drop in cholesterol level by downregulating LDLR, inducing cell death. **B** Representative RXR/LXRα activation in normal neuronal cells **—** stimulated by 9-cis-RA and oxysterol derived from cholesterol, RXR, and LXR heterodimerise to induce the transcription of ATP binding cassette transporter (ABCA1/G1) which is responsible for the efflux of cholesterol from the cell. RXR/LXRα complex induces transcription of inducible degrader of the LDLR (IDOL), ApoE, MerTK, and EEPD1. Cholesterol is imported into the cell through low-density lipoprotein receptors (LDLRs), and fatty acid is imported by CD36. Exogenous ligands honokiol or bexarotene activate RXR in the RXR/PPARγ complex. Further, PPARγ/α is stimulated by the fatty acids and promotes the transcription of ApoA1, CD36, BDNF, and GDNF. Ligand-bound LXRα in RXR/LXRα heterodimer complex can be SUMOylated and inhibit the expression of pro-inflammatory genes such as IL-1β and iNOS

RXR and its interacting partners PPAR and LXR mediate several aspects of lipid metabolism including cellular cholesterol uptake, storage in fat cells, and efflux [18]. LXR agonist LXR-623 treatment of the glioblastoma (GBM) cells was shown to promote cell death [116] (Fig. 3A). The agonist (LXR-623) permeates the blood–brain barrier (BBB) inducing cell degeneration and offers a potential pharmacological treatment for the CNS tumours involving GBM [116]. In monocytes and macrophages, stimulated by agonists such as 9-cis-RA, bexarotene, LG100268, or honokiol, RXR/PPARγ/LXR has been implicated in directly regulating cholesterol efflux by mediating transcription of ABCA1 and ABCG1 [62, 63, 117] (Fig. 3B). PPARγ activation is associated with immune-phenotype manifestations of monocyte-derived dendritic cells through its regulatory effects on genes involved in lipid metabolism, storage, and transport [118]. Moreover, in macrophages, scavenger receptor class B member 3 (SRB1) and the scavenger class A (SR-A) proteins are important receptors for the uptake of various lipid molecules, and their impairment could contribute to atherosclerosis pathology [119, 120] (Fig. 3B). ABCA1 dysfunction in the brain is associated with reduced levels of ApoE [121, 122]. The cholesterol transcytosis between neurons and astrocytes mediated by ApoE, ABCA1, and ABCG1 occurs in an LXR-dependent manner [123]. Cholesterol being one of major components of the myelin sheath is also trafficked by LXRs expressed in the oligodendrocytes [123]. Isotypes of the LXRα/ß are implicated both in demyelination and remyelination as evident from the myelin sheath thinning in the LXR-ablated mice and activation of LXR isoforms by 5-hydroxycholesterol and TO901317 agonists [14, 123]. Due to obligatory heterodimer formation between RXR/LXR, this complex could be a promising therapeutic target in multiple neurological disorders. Both LXR and PPARß/δ in their respective heterodimer complex with RXR coordinate clearance of the apoptotic cells by mediating phagocytosis [124]. In macrophages, fatty acids from the apoptotic cells activate LXR which subsequently activate MerTK and PPARβ/δ [124]. This activation in turn induces C1q and MGFE8 opsonins that facilitate apoptotic cell clearance [124]. In addition, SUMOylation of LXRα in RXR/LXRα complex has been suggested to cause trans-repression of the pro-inflammatory genes by reducing the antibody production [124, 125] (Fig. 3B).

RXRs mediate multiple fatty acid signalling including for DHA following treatment or their dietary intake. These fatty acids are involved in several neural functions including maintaining cognitive integrity and memory-based functions as shown in a study conducted on the BALBcByJ mouse model of schizophrenia [40]. Conversely, DHA and aspirin have also been shown to activate RXRα and PPARα, respectively [126]. DHA inhibited miR-21 and activated PPARα expression in SH-SY5Y cells and, in combination with aspirin, further activated the RXR/PPAR heterodimer leading to increased expression of neurotrophic factors such as brain-derived neurotropic factor (BDNF) and glial cell-derived neurotropic factor (GDNF) and postsynaptic density protein 95 (PSD-95) [126] (Fig. 3B).

### RXR and Its Partners in Neuronal Differentiation and Neuroprotection

**Fig. 4:**
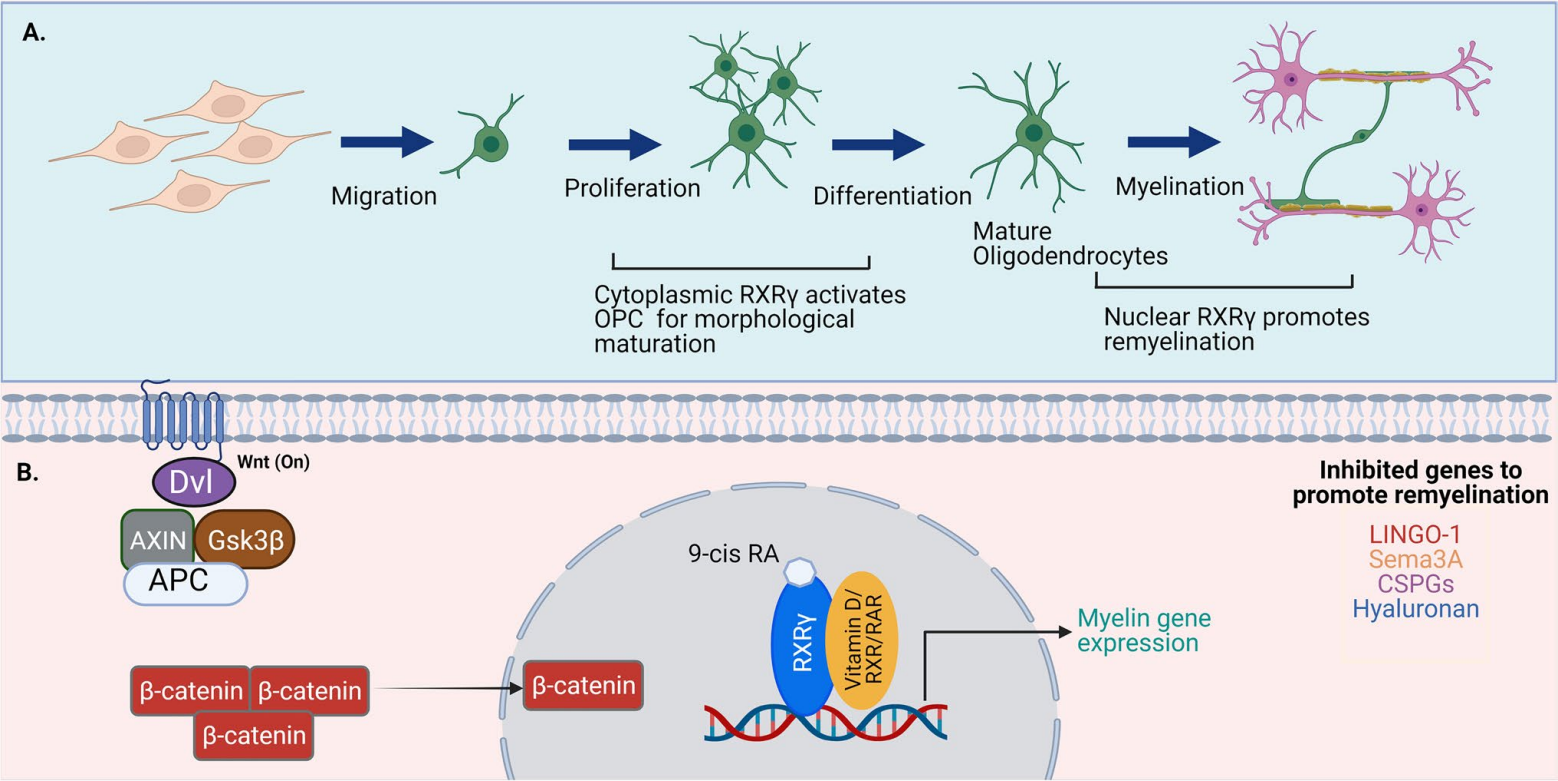
RXR in neuronal differentiation and neuroprotection. **A** RXRγ expressed in the cytoplasm of the oligodendrocyte precursor cells (OPC) plays an important role in oligodendrocyte differentiation and in promoting remyelination. **B** In lesioned tissue undergoing remyelination, RXRγ is localised in the nucleus of the myelinating oligodendrocytes and participate in cell maturation and repair. 9-cis-RA treatment stimulates RXR and together with its dimeric interaction with vitamin D receptors promotes remyelination. RXR and its permissive partners compete against the negative modulators of remyelination LINGO-1, Sema3A, chondroitin sulphate proteoglycan (CSPGs), and hyaluronan to repair neuron following injury. Wnt/β-signalling also plays a role in the regulation of OPC differentiation and in the stimulation of remyelination

Next-generation sequencing approaches deployed to dissect the role of RXR in regulating early neural differentiation and specification have led to the identification of RARγ, LXRβ, and RXRβ as abundantly expressed in undifferentiated embryonic stem cells (ESCs) [127, 128]. Studies have revealed that ligand-dependent activation of RAR/RXR is a dominant inducer of early neurogenesis, while LXR/RXR activation effects are largely limited to early differentiation phases [129]. Accordingly, LXR synthetic agonist GW3965/LG268 demonstrated remarkable impact on cell fate specification of the neuronal progenitors, initiating axonal guidance and neurogenesis [129]. The effects of RXR-mediated signalling on mouse oligodendrocyte differentiation were also suggested by an almost complete differentiation block induced in oligodendrocyte precursor cells that lack RXRγ [130]. In rodents, lysolecithin-induced demyelination of brain slices was associated with the upregulation of RXRγ in the cytoplasm of oligodendrocyte lineage cells [14]. Treatment with 9-cis-RA during remyelination process promoted oligodendrocyte differentiation and myelin repair in RXRγ-dependent manner in vivo [14]. These findings have not only established RXR role in mediating cell differentiation but also highlight RXR functions beyond the nucleus depending on physiological state of the cell [14]. Further, 9-cis-RA-induced RXRγ activation is believed to suppress inhibiting factors of the oligodendrocyte differentiation such as LINGO-1, CSPGs, hyaluronic acid, and Wnt/*β*-catenin signalling resulting in OPC maturation [131] (Fig. 4B). RXR signalling may also be relevant for neuroprotective effects on dopaminergic (DA) neurons. RXR heterodimeric partner Nurr1 is expressed in developing and mature DA neurons in several regions of the brain including the hippocampus and cerebral cortex [11]. Nurr1 and other homologous members NGFIB and Nor1 can be activated by hypoxic/ischemic stress and kainic acid-induced excitotoxicity, and its association with RXR can be neuroprotective for DA neuronal cells [80]. It has been demonstrated that Nurr1 agonist mercaptopurine (6-MP) stimulates Nurr1 by direct binding to its N-terminal AF-1 domain [132]. Treatment with 6-MP was shown to reduce the cerebral infarct in a rodent-permanent middle cerebral artery occlusion (pMCAO) model and lead to reduced inflammatory cytokines IL-1β and TNF-α in the serum and CSF [132]. These observations collectively implicate the role of RXR and its partners in regulating neuronal differentiation and mediating neuroprotective effects.

### RXR and Its Partners in Regulating Glucose Metabolism

**Fig. 5:**
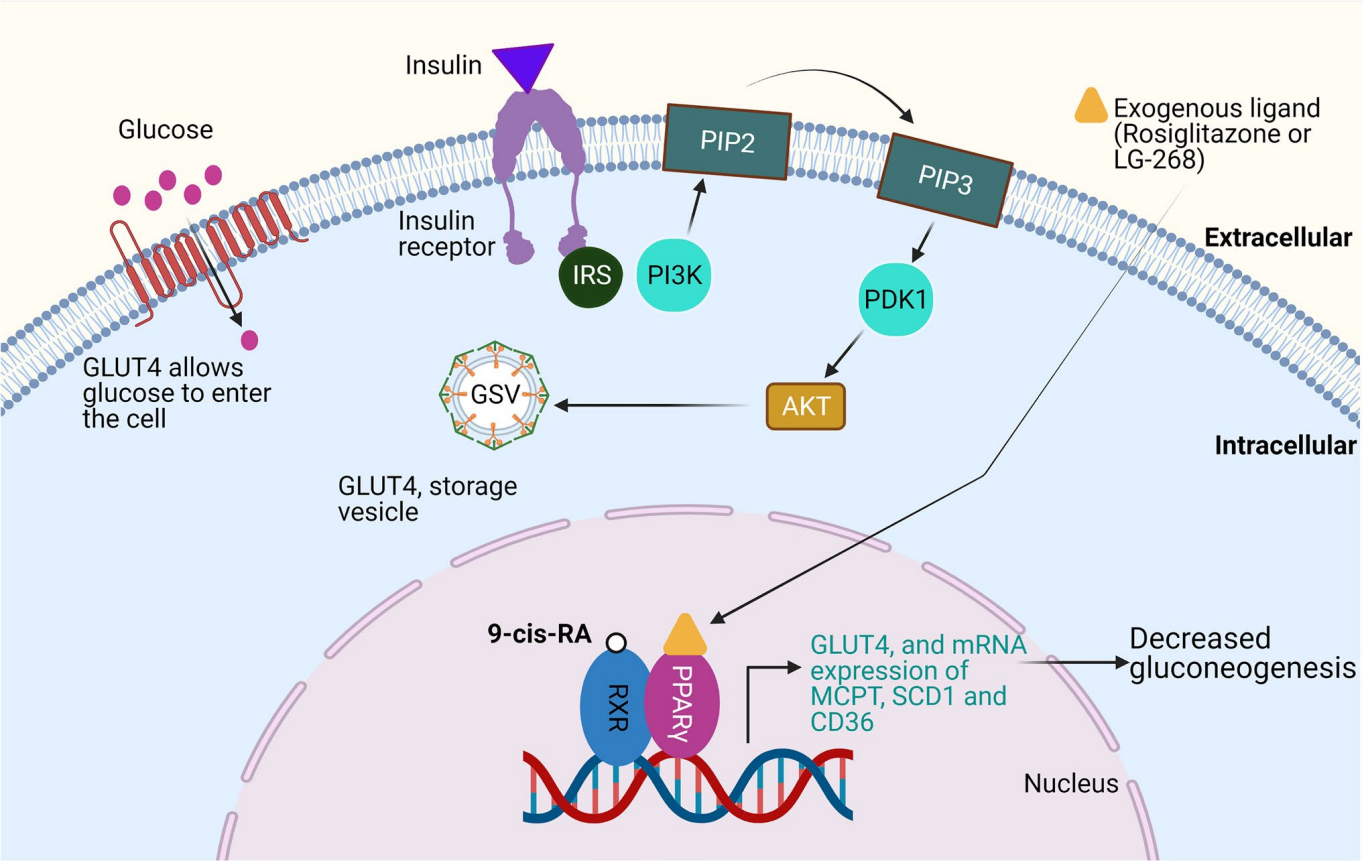
RXR in glucose metabolism: PPARγ/RXR heterodimer complex is activated by synthetic ligand thiazolidinedione, e.g. rosiglitazone, or by 9-cis-RA. This activation upregulates the expression of genes involved in glucose metabolism, e.g. glucose transporter (GLUT4) and mRNA expression of MCPT, SCD1, and CD36. The increased expression of GLUT4 mediates insulin signalling pathway and increases the ability of the cells to take up glucose. Insulin binds to its receptor phosphorylated insulin receptor substrate (IRS) which activates and translocate phosphatidylinositol-3-kinase (PI3K) to the plasma membrane, and PI3K phosphorylates phosphatidylinositol 4,5-biphosphate (PIP2) to phosphatidylinositol-3,4,5-biphosphate (PIP3) and then to AKT. AKT promotes translocation of GLUT4 to cell membrane leading to increased glucose uptake. This process stimulates protein synthesis, glycogenesis, and lipogenesis and decrease gluconeogenesis

RXR via heterodimerisation with PPARγ plays crucial role in mediating glycaemic control and regulating cellular and biochemical effects of insulin actions [78]. 9-cis-RA concentrations in different feeding, fasting, and glucose challenge conditions to the rodents were inversely associated with a decrease in insulin levels [53]. Glucose utilisation by all cell types fluxes through major pathways in excitatory and inhibitory neurons and astrocytes, via glucose transporters GLUT4 and GLUT3 [133, 134]. RXR participates in adipogenesis by regulating early aspects of GLUT4 responsible for linking adipogenesis to subsequent events of lipid metabolism [135]. RXR agonist, LG268, and PPARγ agonist, rosiglitazone, were shown to generate insulin sensitisation in rodent model of type 2 diabetes via overlapping mechanisms [93, 136]. Similar effects of rosiglitazone and LG268 were identified on the metabolic gene expression in white adipose tissue of skeletal muscle and liver in Zucker diabetic fatty rat models [137]. Rosiglitazone activation led to decreased mRNA expression of the TNF-α in adipose tissue [138]. Further, while rosiglitazone treatment induced mRNA expression of GLUT4, mitochondrial carnitine palmitoyl-transferase (MCPT), stearoyl CoA desaturase 1 (SCD1), and CD36, antagonistic or nil effects were observed upon RXR agonist, LG268 treatment [136, 137] (Fig. 5). In contrast, RXR agonist increased the expression of MCPT, SCD1, and CD36 in the liver, whereas rosiglitazone treatment only enhanced the expression of CD36 [136]. This differential regulation of genes in adipose and liver tissues is reflective of the fact that insulin sensitisation may be accomplished by multiple mechanisms [136]. In addition, the anti-diabetic effects of RXR agonist may not simply be a reflection of RXR/PPARγ activation, and that permissiveness with interaction partners can vary within different cell and tissue types [136].

## RXR and Its Partners in Neurological Disorders

**Fig. 6:**
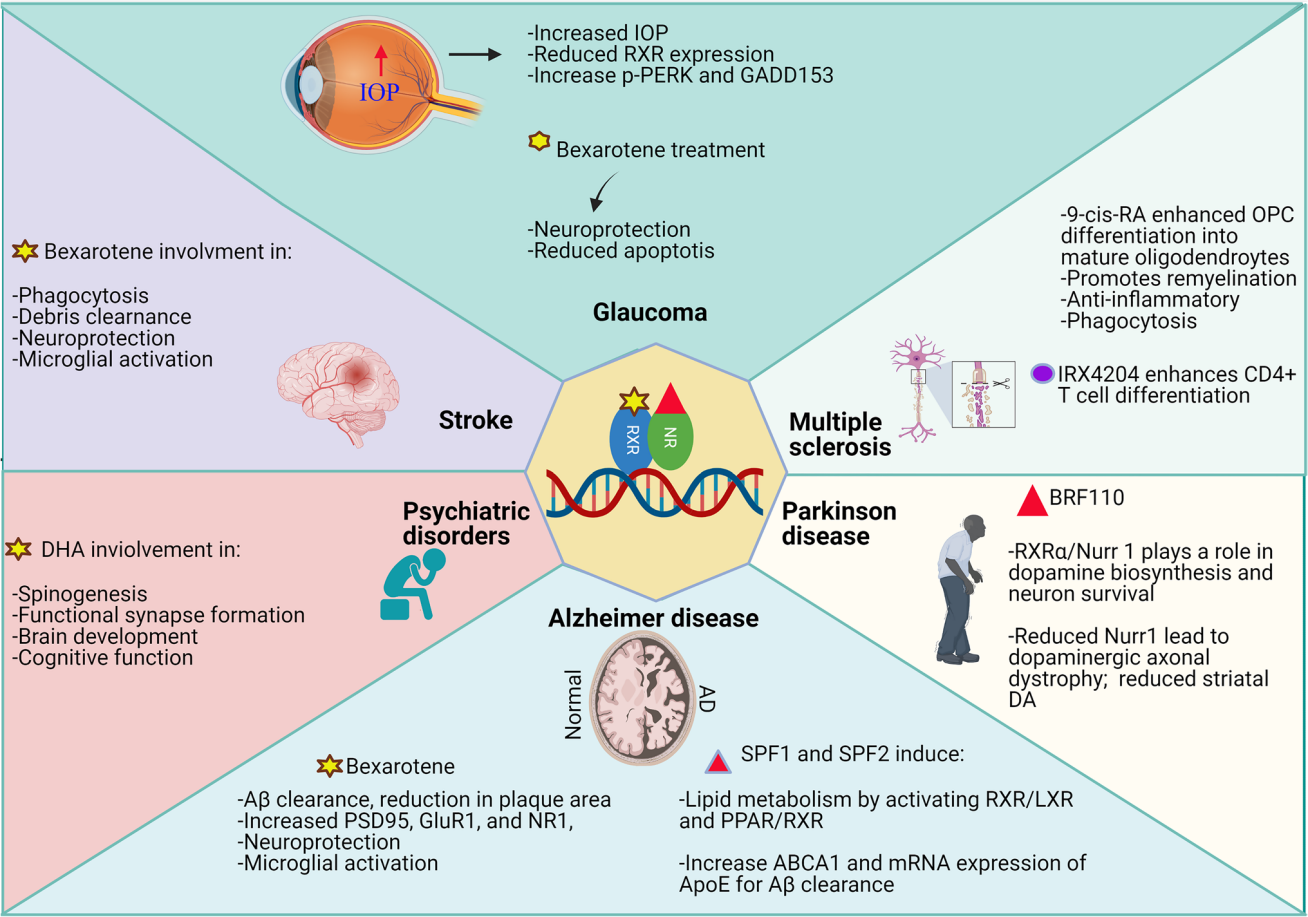
Schematic representation of functional roles of RXR and its heterodimers upon ligand activation in various neurological disorders

Cumulative evidence of RXR roles in mediating Aβ clearance, inducing microglial activation and remyelination in different neurological disorders, has generated interest in greater understanding of the RXR signalling in neuronal tissues [11, 68, 139]. The use of RXR agonists and antagonists may help achieve specific modulation of target genes and biochemical networks, which may serve as a promising therapeutic target [48]. A schematic representation of RXR roles and implications of its direct or indirect activation via heterodimer formation and pharmacological stimulation in neurodegenerative diseases is summarised in Fig. 6.

### Glaucoma

Glaucoma is a one of the leading causes of irreversible vision loss and is characterised by progressive degeneration of the retinal ganglion cells (RGCs) and associated optic neuropathy [140]. RXR α/β/γ isotypes are well expressed in adult ocular tissues including retina, and these receptors also play a critical role in post-natal eye development [141]. Our studies have demonstrated that pharmacological activation of RXR performs a neuroprotective role by rescuing RGCs in murine models of glaucoma [9]. Glaucomatous injury reduced the expression of retinal RXR in both microbead-induced increased intraocular pressure (IOP) model and intravitreal glutamate injection-induced excitotoxicity model [9]. The decrease in RXR expression was particularly evident in the inner retinal layers demonstrated using immunohistochemical analysis, where the decline in all the three RXR isotypes was observed [9]. RXR expression was reinstated by treatment with 100 mg/kg of bexarotene that elicited neuroprotective response in both high IOP and excitotoxicity-induced RGC injury conditions [9]. Bexarotene treatment was also shown to impart protection against ER stress in the retina as evident by the suppression of GADD153 and p-PERK, which are increased in the inner retina in glaucoma [9]. This decrease in ER stress response was concomitant with reduced apoptotic pathway activation in the retina. These findings illustrated that pharmacological activation for RXRs is neuroprotective in the neural retina and prevents functional and structural damage induced by glaucoma injury [9]. Furthermore, HDAC is a constituent of large RXR complex with nuclear receptor corepressor (N-CoR), and studies have indicated that HDAC activity is inversely related to RXR activation caused by bexarotene treatment, in glaucoma models [9]. Proteomics analysis of the retinal and vitreous tissues from the post-mortem eyes from open angle glaucoma and aged-matched control eyes showed enrichment of LXR/RXR and FXR/RXR pathways [142, 143]. LXR, RXR, and FXR are important mediators of the lipid signalling including cholesterol metabolism and inflammation indicating the involvement of these biochemical processes in glaucomatous conditions [144]. The enriched LXR/RXR-mediated network components in mass-spectrometric analysis of glaucoma tissues are suggestive of a potential contribution of RXR in overall pathophysiology of glaucoma.

The effects of RXR have been shown to occur beyond RGCs in the retina and in this regard RXR activation by DHA promoted survival of rat photoreceptors via ERK/MAPK signalling activation during early development [58]. Supporting these observations, RXR pan-agonists HX630 and PA024 rescued photoreceptors from H_2_O_2_-induced apoptosis in retinal cell cultures [58]. Altogether, these studies suggest that RXR signalling is intricately involved in regulating neuronal survival in glaucoma and potentially other retinal disorders. RXR gene polymorphisms have also been suggested to be associated with diabetic retinopathy [145]. A study evaluated 213 diabetes patients of Han Chinese descent, for 9 different RXRγ SNPs, and SNP rs3818569 was found to be enriched in both allele and genotyping frequencies in patients with diabetic retinopathy complications [145]. The exact role of RXRγ SNP in the disease pathogenesis is not established, but the reports suggest potential implications in ischemia-mediated overexpression of cytokines [146]. Genome-wide studies for RXR and its partners will unravel their role in determining genetic predisposition in diabetic retinopathy, glaucoma, and other disorders.

### RXR in Alzheimer Disease

Alzheimer disease (AD) is a devastating neurodegenerative disorder of the CNS that causes debilitating memory loss and extensive deterioration of cognitive and functional abilities [12]. The nuclear receptor superfamily, especially RXR, and its agonists have been investigated for their roles in AD pathology [147]. RXR activation has been shown to exert beneficial effects in AD, and the positive impact of RXR actions can be broadly categorised into those mediating Aβ clearance, suppression of Aβ generation, regulation of neuronal function, and anti-inflammatory actions [12, 148]. Bexarotene treatment in APP/PS1 mouse model of AD was demonstrated to increase clearance of Aβ, which consequently reversed the cognitive impairment caused due to AD pathology [12, 148]. The RXR ligand treatment induced neuronal differentiation and protected cells against age-related synaptic loss through low-density lipoprotein receptor-related protein-1 (LRP1) [149]. However, the protection conferred by bexarotene needs to be interpreted with caution due to cellular toxicity effects associated with the compound [149]. SPF1 and SPF2, prenylated flavonoids isolated from *Sophora tonkinensis gagnep*, were also shown to exhibit selective RXR agonistic activity [68, 150]. These compounds activate RXR/LXR and PPAR/RXR heterodimers and promote ABCA1 and ApoE mRNA expression in PC12 cells. The SPF1 and SPF2 treatment protected the cells against Aβ-induced neurotoxicity by inducing RXR/LXR heterodimerisation [68, 150].

These observations are supported by the reports that retinoic acid isomers including ATRA, 9-cis-RA, and 13-cis-RA induce neuroprotective effects against Aβ-induced cell death [151]. Combination treatment with RARα, *β*-agonist Am80 (tamibarotene), and pan-RXR agonist HX630 significantly improved the memory deficits and suppressed insoluble Aβ peptide concentrations in the AβPP23 mouse model of AD [152]. More recently, thiophene derivative methyl 2-amino-6-(tert-butyl)-4,5,6,7-tetrahydrobenzo[b]thiophene-3-carboxylate (TBTC) which was suggested to function as a preferential agonist of RXRα and is able to induce heterodimerisation of RXRα/LXRα and PPARγ/RXRα was demonstrated to reduce Aβ levels in cell culture as well as in AD animal model [153]. Genistein was similarly shown to enhance clearance of Aβ through PPARγ/ApoE activation in 8-month-old APP/PS1 mice [154].

These beneficial effects of RXR activation were further supported by the findings that RXR expression in APP/PS1 mouse model of AD was inversely associated with Aβ immunogenicity in brain hippocampus lysates [10]. Similar effects were recapitulated in SH-SY5Y neuroblastoma cells where treatment with Aβ peptide (1–42) resulted in reduced RXR expression [10]. RXR agonist bexarotene was able to rescue the loss of the RXR expression in cells after Aβ (1–42) exposure [10]. RXR–bexarotene also reduced ER stress response and supressed apoptosis in neurons [10]. It was illustrated that the low concentrations of bexarotene exerted neuroprotective effects, alleviating ER stress response, and resulted in reduced cellular apoptosis [10]. Treatment with the compound between 0.1 and 1 μM range led to gradual increase in the expression of various RXR isoforms in the cells [10]. In contrast, higher concentration of bexarotene (5–10 μM) promoted ER stress marker GADD153 and p-PERK expression [10]. The increase in ER stress response paralleled with the upregulation of pro-apoptotic BAD protein expression at these concentrations [10]. These findings corroborate observations in human AD studies where bexarotene treatment resulted in reduced Aβ levels in brain and enhanced serum Aβ concentrations, particularly in ApoE4 noncarrier genotype, suggesting that the drug may be acting in ApoE4-dependent manner [155]. Similar effects were observed in 6- and 11-month-old APP/PSEN1 AD mice where bexarotene treatment reduced insoluble Aβ levels and diminished Aβ plaque area by approximately 75%. This corresponded with enhanced ApoE, ABCA1, ABCG1, and HDL levels in the hippocampus and cortex tissues [74]. The beneficial effects of RXR agonist in AD were further evident in 5xFAD mouse model where 2-week bexarotene treatment imparted protection to neuronal cells in subiculum and to the layer V of cortex, rescued memory deficits, and increased pre-and postsynaptic markers, while suppressing neuroinflammation, astrogliosis, and Aβ plaque formation [12]. The drug also promoted Aβ transcytosis across the membrane in vitro model as well as enhanced flux of Aβ across BBB in ApoE ablated mice, inducing clearance of the peptide and reversing cognitive deficits [156].

### Involvement of RXR and Its Interacting Partners in Parkinson Disease Pathology

Parkinson disease (PD) pathology manifests as tremors, rigidity, gait dysfunction, and bradykinesia [157] and is characterised by selective and progressive degeneration of midbrain dopaminergic neurons in substantia nigra (SN), which results in striatal dopamine deficits [158]. Cumulative evidence suggests that pharmacological activation of RXR induces neurotrophic actions in the brain that may impart protective effects on the neuronal cells [80, 159]. Nurr1, a permissive partner of RXR, has emerged as a major target in PD owing to its pivotal role in the survival of the ventral mesencephalic late dopaminergic precursor neurons [160]. In this regard, Spathis and co-workers (2017) designed a synthetic RXRα/Nurr1 agonist BRF110, for pharmacological activation of Nurr1, which can potentially serve as a monotherapy and has shown promising effects in PD models [11]. In contrast, other two RXR agonists, bexarotene and XCT0135908, showed only limited beneficial effects, with XCT0135908 demonstrating low penetrability through the BBB [11]. BRF110 effectively prevented the neuronal loss in SH-SY5Y cells in culture and in Nurr1 heterozygous and transgenic mouse models of the PD in vivo [11]. BRF110 specifically activated the Nurr1/RXRα heterodimers, inducing transcription of the dopamine biosynthesising genes such as tyrosine hydroxylase (TH), aromatic l-amino acid decarboxylase (AADC), and GTP cyclohydrolase I (GCH1) transcription and increased striatal dopamine levels [11]. The drug also protected human-induced pluripotent stem cell-derived dopaminergic neurons against PD-associated damage at 12 μM concentration [11]. Interestingly, a specific RXR agonist IRX4204 induced RXRα/Nurr1 transactivation and promoted TH expressing dopaminergic neuronal survival in ventral midbrain neuronal cells in a dose-dependent manner [161]. The oral administration of the compound at 10 mg/kg/day resulted in achieving brain tissue concentration of 11.5 ± 2.9 nM and translated to diminished PD behavioural phenotype while protecting against DA neuronal loss in a rat model of PD [161]. The treatment also promoted the transcription of neurotropic factor GDNF, suggesting possible avenues for the combinatory therapy in PD [161]. More recently, α-synuclein (α-Syn), key protein implicated in PD, has been shown to cross-talk with RXR/RAR and PPARγ-induced gene transcription [162]. α-Syn was demonstrated to bind to the retinoic acid for translocation to the nucleus in SH-SY5Ycells and induce transcriptional activation [163]. The transcription analysis of SH-SY5Y induced with doxycycline revealed that the nuclear receptors that are activated with retinoic acid were also activated upon α-Syn/retinoic acid treatment [163]. The transcription activities associated with RXRα, RARβ, PPARγ, LXR, and THR were observed to be enhanced by α-Syn/retinoic acid exposure [163]. These findings are mainly derived from in vitro studies, and future in vivo studies will further establish the relevance of these results to the PD pathophysiology [163].

Additionally, RXR and its heterodimeric partner Nur77 have been implicated in the homeostatic regulation of the dopaminergic system, which in addition to PD is intricately linked to schizophrenia, autism, bipolar disorder, depression, and addiction regulation [164, 165]. Mutations in RXRβ (Val95Ala), PPARα (Val227Ala), and nuclear-related receptor 1 (EX8 + 657(CA)9‐10) have been identified in neuropsychiatric patients [166]. Further research will elucidate whether pharmacological or genetic regulation of RXR signalling is beneficial in preserving mental health resilience.

### Participation of RXR Biochemical Network in Multiple Sclerosis Neuropathology

Multiple sclerosis (MS) is a heterogeneous neurological disorder characterised by inflammatory changes accompanying progressive demyelination and axonal loss [167]. The disease is associated with transient episodes of autoreactive CD4 + T cell-mediated inflammatory attacks resulting in loss of myelin sheath and axonal dysfunction [168]. RXR along with its permissive heterodimeric partners plays crucial role in immune cell regulation, inflammation, and oligodendrocyte lineage progression which are important aspects of MS neuropathology [169]. RXRα is expressed by T cells and other haematopoietic cell types that extensively participate in inflammatory responses [170]. Animal models of demyelination demonstrate increased RXRγ expression in oligodendrocyte lineage cells that are involved in remyelination in MS lesions [14]. This suggests a biochemical compensation by oligodendrocytes to resist the demyelination effects [14]. Blockade of RXR signalling achieved through targeted mRNA silencing, pharmacological antagonism, or null mutation of RXRγ revealed inhibition of OPC differentiation and remyelination, but no effects on OPC survival were observed, indicating that RXR signalling may specifically be associated with biochemical mechanisms involved in cell differentiation and myelination [130, 169]. These findings were supported by observations that RXR promoted differentiation of OPCs into mature oligodendrocytes in experimental autoimmune encephalomyelitis (EAE) animal model [171]. Treatment with 9-cis-RA resulted in RXRγ activation and enhanced the differentiation of OPCs into mature oligodendrocytes [14].

Further, RXR signalling is believed to play a vital role in the phagocytic removal of myelin debris by monocytes and macrophages [139]. Myelin fragments produced during demyelination process may inhibit OPC differentiation, thus impairing the remyelination process [139]. Cellular capacity for phagocytic removal of myelin debris decreases with ageing which is also associated with diminished expression of RXR in monocytes and macrophages. Activation of RXR with 9-cis-RA was shown to enhance phagocytosis in ageing macrophages [139]. Bexarotene treatment significantly increased phagocytosis of myelin debris in monocytes in lysolecithin-induced focal demyelination mouse model [139]. Thus, RXR activation may both enhance the OPC differentiation and maturation, as well as induce clearance of myelin debris by monocytes, macrophages, and microglia, processes that are favourable to promote remyelination in MS [131]. Activation of RXR has been shown to supress pathological symptoms observed in animal models of MS [172]. 9-cis-RA-mediated activation suppressed astrocytic and microglial pro-inflammatory responses in vivo [173], and drug was shown to reduce the severity of disease in EAE, model [174]. Another highly selective and potent RXR agonist, IRX4204, also demonstrated enhanced differentiation of CD4 + T cells into inducible regulatory T cells (iTreg) while restricting the differentiation of pro-inflammatory T helper (Th)-17 cells in vitro and effectively attenuated the severity of disease in EAE model [174–176]. Thus, RXR may exert a multipronged influence in MS as receptor signalling plays significant roles in both immunomodulation and remyelination pathways.

### Involvement of RXR and Its Heterodimers in Stroke

RXR is implicated in orchestrating microglial phagocytic functions in the brain and mediate clearance of apoptotic neuronal debris [139]. Macrophages and microglial cells play key roles in removing cellular fragments and protein aggregates that are formed during cellular degeneration and protect against tissue atrophy caused by ischaemic as well as haemorrhagic stroke episodes [13]. As mentioned previously, RXR is widely expressed in macrophages and plays a role in fine-tuning the transcription of several genes [104]. In the microglial cells derived from mouse brain, bexarotene treatment enhanced ApoE, ABCA1, and SREBF2 transcription, which are involved in lipid metabolism, as well as CD36, AXL, MerTK, and TGM2 genes that play key roles in regulating phagocytosis and mediating recovery of damaged tissues [119, 125]. Intriguingly, microglia derived from myeloid cell-specific conditionally ablated RXRα mice demonstrated only limited effects of bexarotene on transcriptional activation of the above-mentioned genes establishing pivotal role of RXR isoform in regulating these biochemical pathways [13]. Further investigations established that microglial and macrophagic ability to induce phagocytosis was impaired upon RXRα ablation, and while bexarotene promoted phagocytic capacity in control cells, the effects were not evident in cells derived from RXRα ablated mice [13]. These findings paralleled with reduced expression of bFGF and VEGF, genes that are implicated in cell/tissue repair and macrophage/microglial markers CD36, CD163, CD204, CD206, and ABCA1 that determine the scavenging function of immune cells, in RXRα impaired cells derived from ischemic mouse brain [13]. Phenotypically RXRα-ablated mice depicted significantly greater brain atrophy and neurological deficits compared to the control animals [13]. Bexarotene treatment was able to rescue the post-stroke recuperation in control but not in the RXRα-ablated animals [13]. Together, these observations establish key role of RXRα in determining the stroke-mediated damage and beneficial effects of pharmacological modulation of RXR in imparting protection against these deficits [13].

Further, Nurr1 heterodimerisation with RXR has been shown to confer neuroprotection and provide anti-inflammatory effects through NF-κB inhibitory actions in middle cerebral artery occlusion ischemic stroke model [177]. Nurr1/RXR agonist HX600 protected against motor impairment in mice and reduced microglial pro-inflammatory mediators preventing inflammation-induced neuronal death in co-cultured neuronal and microglia in vitro model [177]. Administration of HX600 to ischemia mouse model resulted in the downregulation of Iba1, p38, and TREM2 markers and reduced lyso-phosphatidylcholine and acylcarnitine lipid metabolites [177]. The treatment was also effective in imparting protection to endogenous microglia from ischemia-induced apoptosis and prevented leukocyte infiltration into the tissue [177]. Nurr1/RXR may therefore provide novel mechanism-based targets in addressing the neuroinflammatory aspects in stroke.

## Clinical Trials Focussing on Use of RXR and Its Agonists

RXR and its partners are being investigated in several clinical trials for use as a potential drug target in AD and PD. A look at the (https://clinicaltrials.gov/ as of 6th August 2021) registry illustrates 20 clinical trials that are listed in the repository, with status as either completed (11), currently recruiting (3), or terminated (2), with the remainder as not recruiting or are with unknown status. Amongst these, a phase 2 clinical trial conducted by Cleveland Clinic (NCT01782742, Las Vegas, Nevada) evaluated the efficacy and safety of RXR agonist bexarotene in patients suffering from mild to moderate AD. The primary outcome of the Brain Energy for Amyloid Transformation in AD or BEAT-AD study was measured using 18F-AV-45 positron emission tomography (PET) imaging of brain for Aβ load. No changes in brain amyloid load were observed in ApoE carriers, while ApoE non-carriers depicted a decrease in brain amyloid load [148]. However, the overall outcome of this study was negative, and even though bexarotene reduced Aβ levels in ApoE non-carriers, it resulted in elevated triglycerides presenting a cardiovascular risk [148].

Another study sponsored by ReXceptor, Inc. (NCT02061878, Orlando, Florida) aimed to determine whether bexarotene treatment in humans alters the CSF levels of ApoE or clearance of Aβ peptide [155]. The study only included 12 participants, and the results were not published in the public domain [155]. Next, a phase 1 clinical trial sponsored by Lo therapeutics (NCT02438215, Molecular NeuroImaging, New Haven, Connecticut) on RXR agonist IRX4204, a potent and selective BBB penetrant, was conducted to examine dopamine transporter density using [123I] *β*-CIT single-photon emission tomography (SPECT) before and following treatment with drug [178]. Changes in motor and cognitive parameters were also studied for a 30-day period in 15 early PD subjects in this study [178]. The study indicated safe use and tolerance at dosage of 5 and 10 mg/day for 30 days with no short-term effects on the dopamine transporter binding [178]. We anticipate that results of at least some of these studies will help design further large-scale randomised clinical trials on drugs targeting RXR effects in the neurological disorders. A potential limitation is that different regions of the brain and various cell types can be affected differently and whole-brain imaging may not be able to detect subtle changes induced by RXR agonist treatment.

## Concluding Remarks and Future Perspectives

RXRs and their interacting partners play a pivotal role in regulating cellular and biochemical processes relevant to neuronal health, and this positions the receptor family members and their downstream signalling as a potential therapeutic target in multiple neurological disorders. RXR activation is instrumental in fine-tuning the transcription of several genes which means that these receptors mediate critical nodes that lie at the crossroads of key biochemical networks. Alterations in expression of various RXR isoforms, their downstream signalling, and regulatory molecules including their endogenous ligands have been detected in disease conditions. Studies focussing on the activation of RXR via 9-cis-DHRA and DHA ligands can potentially provide mechanistic insights into specific functioning of RXR isotypes in CNS. There is evidence to indicate that RXR activation in heterodimeric complex enhances the expression of neurotrophic factors such as BDNF and GDNF, which have established neuroprotective roles [126]. Thus, it is likely that a proportion of RXR-induced effects is mediated through its influence on bolstering expression of neurotrophic factors in CNS. RXR and its heterodimeric partners, however, have been implicated in regulating host of additional biochemical and metabolic responses such as glucose and cholesterol metabolism and in maintaining overall neuronal physiology and may have effects independent of neurotrophic factor mediated actions.

Studies in transgenic and knockout animal models have indicated that global ablation of the RXRα^−/−^ results into an embryonic lethal phenotype; however, compound RXRα^+/−^, RXRβ^−/−^, and RXRγ^−/−^ knockout mice survive and can be utilised to investigate development related and functional roles of these receptors in vivo [179]. Several interacting partners of RXR have been identified in the recent years, and future investigations will facilitate identification of the novel cell and organelle-specific partners of RXR in health and disease conditions. While traditionally immune precipitation-based methods have been used to identify the interacting partners, mass spectrometry-based proteomics methods coupled with ChIP assay will provide a high-throughput approach to determine novel protein–protein and DNA–protein interactions. Further, RXR-focussed computational and bioinformatic studies will help identify novel and more specific ligands that could rationalise drug development. This could also help in the development of synthetic and naturally occurring ligands leading to improved BBB permeability. A significant limitation in RXR research arises from its biochemical and functional redundancy with other nuclear receptor family members. This coupled with overlapping ligand recognition and transcription activation adds to the complexity to understand the RXR biology in a cell- and tissue-specific manner. Designing or identification of specific ligands for individual RXR isotypes will help understand receptor mechanism as well as develop CNS targeted treatment strategies.

## Data Availability

Not applicable.

## Abbreviations

APP: Amyloid precursor protein
Akt: Protein kinase B
AXL: A receptor tyrosine kinase
BAD: BCL2-associated agonist of cell death
bFGF: Basic fibroblast growth factor
C1q: Complement component 1q
CD36: Cluster of differentiation 36
ChIP: Chromatin immunoprecipitation
CRBPII: Cellular retinol binding protein type II
CYP19: Cytochrome P450, family 19
EEPD1: Endonuclease/exonuclease/phosphatase family domain-containing protein 1
eNOS: Endothelial nitric oxide synthase
FoxO3A: Forkhead box protein O3
GADD153: Growth arrest and DNA damage 153Ib-1a: ionised calcium-binding adapter molecule
IL-1β: Interleukin-1β
iNOS: Inducible nitric oxide synthase
LDLR: Low-density lipoprotein receptor
LINGO: Leucine-rich repeat and immunoglobin-like domain-containing protein 1
MAPK: Mitogen-activated protein kinase
MerTK: Myeloid–epithelial–reproductive protein tyrosine kinase
MGFE8: Milk fat globule epidermal growth factor
MnSOD: Manganese superoxide dismutase
NF-κB: Nuclear factor kappa light chain enhancer of activated B cells
p-PERK: Phosphorylated extracellular-signal-regulated kinase
PGC-1: Peroxisome proliferator-activated receptor gamma coactivator 1
PS1: Presenilin1
PTX3: Pentraxin 3
Sema3A: Semaphorin 3A
SIRT6: Sirtuin 6
SNP: Single-nucleotide polymorphism
SREBF2: Sterol regulatory element binding transcription factor 2
STAT3: Signal transducer and activator of transcription 3
TGM2: Tissue transglutaminase
TREM2: Triggering receptor expressed on myeloid cells 2
UCP2: Uncoupling protein 2
VEGF: Vascular endothelial growth factor
