# Correction to: Retinoid X Receptor: Cellular and Biochemical Roles of Nuclear Receptor with a Focus on Neuropathological Involvement

**DOI:** 10.1007/s12035-022-02767-w

**Published:** 2022-03-12

**Authors:** Samridhi Sharma, Ting Shen, Nitin Chitranshi, Veer Gupta, Devaraj Basavarajappa, Soumalya Sarkar, Mehdi Mirzaei, Yuyi You, Wojciech Krezel, Stuart L. Graham, Vivek Gupta

**Affiliations:** 1 Macquarie Medical School, Faculty of Medicine, Health and Human Sciences, Macquarie University, Sydney, NSW Australia; 2 School of Medicine, Deakin University, Melbourne, VIC Australia; 3 Save Sight Institute, University of Sydney, Sydney, NSW Australia; 4 Institut de Genetique Et de Biologie Moleculaire Et, Cellulaire, INSERM U1258, CNRS UMR 7104, Unistra, 67404 Illkirch, Graffenstaden France

**Correction to: Molecular Neurobiology**

10.1007/s12035-021-02709-y

In the original manuscript, the author "Soumalya Sarkar" was accidentally deleted during manuscript processing. The article is corrected to add "Soumalya Sarkar" as 6^th^ author of this manuscript.

The orignal paper has been corrected.

